# Primary Choledocholithiasis 15 Years Postcholecystectomy

**DOI:** 10.1155/2020/3265010

**Published:** 2020-10-26

**Authors:** Michael Simon, Irfan Nazir Hassan, Dhanasekaran Ramasamy, David Wilson

**Affiliations:** ^1^Department of Radiology, Saint Barnabas Medical Center, 94 Old Short Hills Road, Livingston, NJ 07039, USA; ^2^Department of Gastroenterology, Saint Barnabas Medical Center, 94 Old Short Hills Road, Livingston, NJ 07039, USA

## Abstract

Gallstone disease is extremely prevalent in the western society with laparoscopic cholecystectomy (LC) being the standard treatment for patients with symptomatic gallstones. The prevalence of common bile duct (CBD) stones with concomitant gallstones increases with age from 8–15% in patients <60 years of age and up to 60% in the elderly. There have been only a few case reports of postcholecystectomy bile duct stones occurring more than 10 years following surgery in the literature. Most of these reports describe the presence of stones within the gallbladder/cystic duct remnant or secondary to migrating surgical clips.

## 1. Introduction

Gallstone disease is extremely prevalent in the western society with laparoscopic cholecystectomy (LC) being the standard treatment for patients with symptomatic gallstones. The prevalence of common bile duct (CBD) stones with concomitant gallstones increases with age from 8–15% in patients <60 years of age and up to 60% in the elderly [1]. For patients suspected of having CBD stones, endoscopic retrograde cholangiopancreatography (ERCP) plays an important role preoperatively. Most patients experience relief of symptoms after LC, but a small number experience postcholecystectomy syndrome, which presents as biliary colic [2]. Postcholecystectomy syndrome can be secondary to dysfunction of the sphincter of Oddi, traumatic stricture, retained CBD stones or retained stones within a gallbladder remnant [2]. The condition is especially common within the first three years postprocedure [3]. There have been only a few case reports of postcholecystectomy bile duct stones occurring more than 10 years following surgery in the literature. Most of these reports describe the presence of stones within the gallbladder/cystic duct remnant or secondary to migrating surgical clips [4, 5].We report a unique case of intra- and extrahepatic choledocholithiasis 15 years following laparoscopic cholecystectomy secondary to a primary biliary stone.

## 2. Case

An 86-year-old male presented to the Emergency Department (ED) with complaints of abdominal pain. The patient's medical history included hyperlipidemia and cholecystectomy 15 years prior to presentation. The patient stated that he had eaten at a fast food restaurant one day prior to presentation. Afterwards, the patient developed mild, diffuse, constant abdominal pain with nausea that worsened on the day of presentation, prompting his ED visit.

In the ED, the patient had a temperature of 102.3°F, leukocytosis with left shift, and a positive urinalysis. In addition, the patient was noted to have hyperbilirubinemia to 4.5 with elevated liver enzymes (ALT 413, AST 340, and ALP 203).

**Figure 1:**
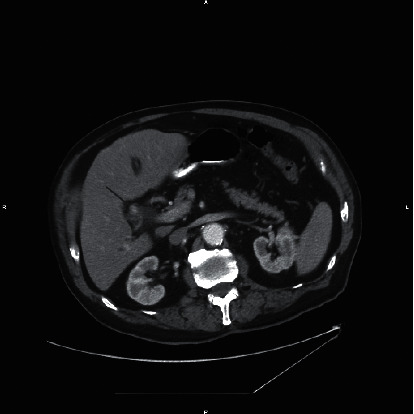
Axial CT of the abdomen with contrast at the level of the junction of the right and left hepatic ducts demonstrates an 11 mm hyperdensity likely representing a calculus (arrow). This finding is confirmed on the coronal image (arrow) in Figure 2.

**Figure 2:**
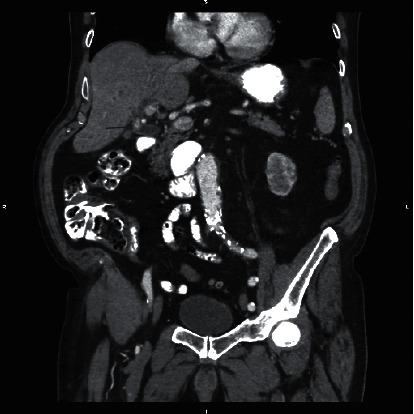
Coronal CT of the abdomen with contrast demonstrates 11 mm hyperdensity likely representing a calculus at the origin of the common bile duct (arrow).

**Figure 3:**
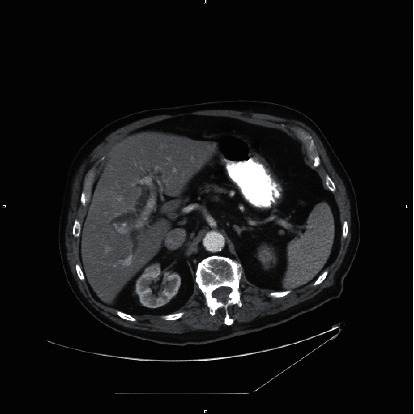
Axial CT of the abdomen with contrast demonstrates intrahepatic biliary dilatation.

A CT scan of the abdomen and pelvis with oral and intravenous contrast demonstrated dilated intrahepatic and extrahepatic biliary ducts with the common bile duct measuring up to 12 mm. There were multiple hyperdensities within the bile ducts particularly at the junction of the right and left hepatic ducts in addition to the distal CBD, consistent with choledocholithiasis (Figures 1–3).

**Figure 4:**
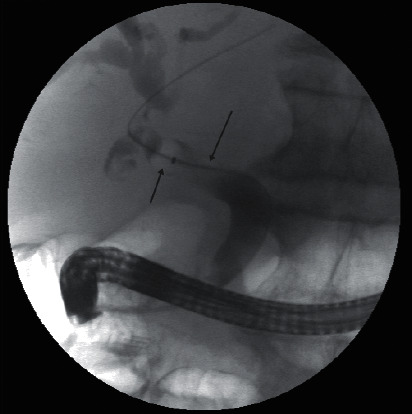
ERCP images demonstrate multiple filling defects (green arrows) within the common bile duct with moderate dilatation of the extra- and intrahepatic biliary tree. A complete sphincterotomy was performed, and multiple black pigmented stones were removed.

An ERCP confirmed the presence of stones within the bile ducts with multiple pigmented black stones removed (Figure 4). Pus was also noted and sent for culture confirming the presence of cholangitis. A 10 Fr stent was placed into the common bile duct due to cholangitis.

The patient tolerated the procedure well and was successfully treated with antibiotics and discharged home. The patient was asked to follow-up with his gastroenterologist as an outpatient.

## 3. Discussion

Choledocholithiasis refers to the presence of bile duct stones. It is estimated that up to 15% of patients <60 years of age have stones in the common bile duct, whereas it can be as high as 60% in the elderly [1]. Risk factors identified for complicated choledocholithiasis are Asian descent, increasing age, and male gender [6, 7]. It is estimated that between 3.4 and 10% of patients have choledocholithiasis at the time of cholecystectomy [8, 9]. Delayed choledocholithiasis has been reported in patients with the most common etiologies being retained or regeneration of stones within a gallbladder remnant or cystic duct [4, 8].

Studies have shown that the vast majority of patients (80%) with choledocholithiasis following cholecystectomy will present within 3 years of surgery [3, 9]. Presentation later than this is thought to be secondary to migrating surgical clips with cases being reported up to twenty years following cholecystectomy. Only a few reports have identified disease at a later interval [10]. Rarely were the stones deemed to be primary as in our case.

Intrahepatic lithiasis can be either primary or secondary. Primary intrahepatic lithiasis is less common in the US and is usually the result of single or multiple cystic dilatations of the intrahepatic biliary tree resulting bile stasis. Secondary intrahepatic stones are the result of migration of gallbladder or CBD stones or strictures related to previous surgery [11]. Other risk factors include prior sphincterotomy, which could allow for debris to enter the CBD leading to infection and nidus formation and metabolic disorders.

Saharia et al. described a group of 30 patients with primary biliary stones following cholecystectomy with an average of 12 years following surgery until diagnosis. The most frequent presenting symptom was acute cholangitis as in our patient [12]. This was one of the few articles that were found after extensive research describing primary choledocholithiasis following cholecystectomy.

## 4. Conclusion

We present a rare case of primary choledocholithiasis postcholecystectomy, which, to our knowledge, is one of only a few in literature studies. Given the latency of presentation, it is important to have choledocholithiasis in mind even a number of years after cholecystectomy.

## Data Availability

All information regarding the patient can be found in the hospital EMR and PACS.
